# Supplementary material for: Validation of Health Education Material for Youth: A Step to Ensure Implementation Fidelity in Community-Based Interventions
Source: Healthcare (Basel). 2019 Dec 27;8(1):8. doi: 10.3390/healthcare8010008 (PMC7151179; doi:10.3390/healthcare8010008)
Supplement: Supplementary file 1 [file healthcare-08-00008-s001.pdf]

Supplementary Table S1. Questions for pre- and post-test knowledge assessment (construct validity) in the questionnaires for adolescent boys and girls (n = 25).

Four sections	Questions in each section for adolescent boys and girls
Reproductive section	Q1. Pubertal changes in girls or boys<br>Q2. Truth about menstruation (girls) or nightfall (boys)<br>Q3. What do irregular periods (girls) or masturbation in boys indicate?<br>Q4. Physiology of menstruation (girls) or how important is size of penis (boys)?<br>Q5. Age at menarche (girls) or age at puberty in boys<br>Q6. Duration of a menstrual cycle or what is semen (boys)?<br>Q7. Importance of genital hygiene<br>Q8. Truth about emergency contraceptives<br>Q9. Routes of transmission of HIV infection between male and female Q10. Is AIDS curable?<br>Q11. Truth about AIDS<br>Q12 Contraceptives that are protective against HIV/AIDS
Mental health section	Q13. Symptoms of mental problems during adolescence<br>Q14. Life skills required for prevention of drug addiction<br>Q15. Psychological changes during puberty
Nutrition section	Q16. What does deficiency of iron leads to?<br>Q17. Fruits are rich in which micronutrients?<br>Q18. Lifestyle risk factors for non-communicable diseases<br>Q19. Side effects of drinking and smoking
Miscellaneous section	Q20. Hand hygiene<br>Q21. Side effects of early marriage<br>Q22. First aid steps<br>Q23. Action after dog bite<br>Q24. Truth about intimate partner violence<br>Q25. Legal age of marriage

Supplementary Table S2. Questions for pre- and post-test knowledge assessment (construct validity) in the questionnaire for young married women (n = 19).

Three sections	Questions in each section for young married women
Reproductive section	Q1. Exclusive breastfeeding<br>Q2. Maternal nutrition during pregnancy<br>Q3. HIV tests of women during pregnancy<br>Q4. When should a woman go for antenatal check-ups first time?<br>Q5. Minimum number of antenatal check-ups needed<br>Q6. Responsibility of family planning<br>Q7. Types of contraceptives<br>Q8. Abortion care<br>Q9. Routes of HIV transmission<br>Q10. Do mosquitoes spread HIV?<br>Q11. Contraceptives protective of HIV
Preconception care section	Q12. Should a woman get herself tested during pre-conception period for diabetes, hypertension, anemia, sexually transmitted diseases?<br>Q13. Should women test herself for familial or genetic diseases before pregnancy?<br>Q14. Effects of violence during pregnancy on child<br>Q15. Three types of food groups<br>Q16. Which foods are rich in calories or energy?
Miscellaneous section	Q17. Government of India scheme for empowerment of women<br>Q18. Women helpline number<br>Q19. Legal age of marriage

Supplementary Table S3. Session's name in the two flipbooks (*Yauvan* and *Sangini*).

Session sequence	Name of the sessions in '*Yauvan flipbook*' for adolescents	Name of the sessions in '*Sangini flipbook*' for young married women
Session 1	Life skills	Women empowerment
Session 2	Pubertal changes	Marital communication
Session 3	Nutrition care during adolescence	Pre-conception care
Session 4	Lifestyle disorders	HIV/AIDS prevention and reproductive health
Session 5	Trauma and injuries	Family planning and abortion
Session 6	Violence among adolescents	Pregnancy care
Session 7	Gender based violence	Healthy relationships and domestic violence
Session 8	Mental health	Post-partum care
Session 9	Substance abuse and stress management	Nutritional care
Session 10	Sexual and reproductive health	Lifestyle disorders
Session 11	Child marriage prevention	Men's health
Session 12	AIDS/Sexually transmitted diseases	Mental health

Supplemenatry Table S4. Evaluation of relevance of agreement on the sessions, stories, photos and key messages of the flipbooks.

S.no.	Item (*Sangini Flipbook*)	Relevance* (n=42) N (I)	Item (*Yauvan Flipbook*)	Relevance* (n=42)N (I)
1.	A session on women empowerment	42 (1.00)	A session on life skills	41 (0.97)
2.	A session on marital communication	41 (0.97)	A session on pubertal changes	42 (1.00)
3.	A session on pre-conception care	41 (0.97)	A session on nutrition care during adolescence	42 (1.00)
4.	A session on HIV/AIDS prevention and reproductive health	41 (0.97)	A session on lifestyle disorders	38 (0.97)
5.	A session on family planning and abortion	40 (0.95)	A session on trauma and injuries	39 (0.93)
6.	A session on pregnancy care	41 (0.97)	A session on violence among adolescents	41 (0.97)
7.	A session on healthy relationships and domestic violence	40 (0.95)	A session on gender based violence	41 (0.97)
8.	A session on post-partum care	41 (0.97)	A session on Mental health	40 (0.95)
9.	A session on nutritional care	42 (1.00)	A session on substance abuse and stress management	40 (0.95)
10.	A session on lifestyle disorders	39 (0.93)	A session on Sexual and reproductive health	39 (0.93)
11.	A session on men's health	40 (0.95)	A session on child marriage prevention	41 (0.97)
12.	A session on mental health	41 (0.97)	A session on AIDS/STD	40 (0.95)
13.	Key messages in the end	40 (0.95)	Key messages in the end	40 (0.95)
14.	Pictures or photos in each session	37 (0.88)	Pictures or photos in each session	37 (0.88)
15.	Stories at the end of session	40 (0.95)	Stories at the end of each session	39 (0.93)
	Mean index score	0.96		0.95

* Number of participants agreed to the relevance of each item; **I: Index.
